# Isolation and characterization of two newly established thymoma PDXs from two relapses of the same patient: a new tool to investigate thymic malignancies

**DOI:** 10.1186/s13046-022-02554-4

**Published:** 2022-12-14

**Authors:** Paolo Mendogni, Roberta Affatato, Enrico Cabri, Michela Chiappa, Gloriana Ndembe, Davide Tosi, Alessandro Del Gobbo, Maddalena Fratelli, Eleonora Pardini, Iacopo Petrini, Lorenzo Rosso, Massimo Broggini, Mirko Marabese

**Affiliations:** 1 Thoracic Surgery and Lung Transplant Unit, Fondazione IRCCS Ca’ Granda-Ospedale Maggiore Policlinico, 20122 Milan, Italy; 2 Laboratory of Molecular Pharmacology, Istituto di Ricerche Farmacologiche Mario Negri IRCCS, 20156 Milan, Italy; 3 Department of Biochemistry, Istituto di Ricerche Farmacologiche Mario Negri IRCCS, 20156 Milan, Italy; 4 Division of Pathology, Fondazione IRCCS Ca’ Granda-Ospedale Maggiore Policlinico, 20122 Milan, Italy; 5 Department of Translational Research & New Technologies in Surgery and Medicine, University of Pisa and Azienda Ospedaliero Universitaria Pisana, 56100 Pisa, Italy

**Keywords:** Thymoma, Patient-derived xenograft, Pre-clinical model

## Abstract

**Background:**

Thymic malignancies are a heterogeneous group of rare cancers for which systemic chemotherapy is the standard treatment in the setting of advanced, recurrent or refractory diseases. Both environmental and genetic risk factors have not been fully clarified and few target-specific drugs have been developed for thymic epithelial tumors. A major challenge in studying thymic epithelial tumors is the lack of preclinical models for translational studies.

**Main body:**

Starting from bioptic material of two consecutive recurrences of the same patient, we generated two patient-derived xenografts. The patient-derived xenografts models were characterized for histology by immunohistochemistry and mutations using next-generation sequencing. When compared to the original tumors resected from the patient, the two patient-derived xenografts had preserved morphology after the stain with hematoxylin and eosin, although there was a moderate degree of de-differentiation. From a molecular point of view, the two patient-derived xenografts maintained 74.3 and 61.8% of the mutations present in the human tumor of origin.

**Short conclusion:**

The newly generated patient-derived xenografts recapitulate both the molecular characteristics and the evolution of the thymoma it derives from well, allowing to address open questions for this rare cancer.

**Supplementary Information:**

The online version contains supplementary material available at 10.1186/s13046-022-02554-4.

## Background

Thymic epithelial tumors (TETs) are a heterogeneous group of rare cancers, with an annual crude incidence rate between 2 and 3.5 per million individuals. Since survival is quite long, the prevalence of TETs is not negligible despite their rarity [1].

According to the World Health Organization (WHO) classification, TETs include two different entities: thymoma, with the subgroups A, AB, B1, B2, B3, and thymic carcinoma. The most common thymoma subtype is B2 (4-46%), followed by AB (11-43%), B1 (8-38%), B3 (6-34%) and A (5-24%). Thymic carcinoma has a lower incidence than thymoma (< 0.1 per 1,000,000 individuals /year) and more aggressive behavior, with worse survival (13% vs 65% at 5 years) [2]. Thymoma A and AB usually have indolent behavior and are mainly diagnosed in early stages. Thymomas B1, B2 and B3 are more aggressive, B3 thymomas having the greatest tendency to intrathoracic spread. Thymic carcinoma is an aggressive tumor, with distinct biological behavior characterized by common lymphatic and hematogenous spread [3].

Surgery is the mainstay of curative-intent treatment: complete resection offers the most significantly favorable prognostic factor for overall survival. When the patient is not deemed to be a surgical candidate, systemic chemotherapy is the standard. Cisplatin-based combination regimens with anthracyclines and/or etoposide are standard of care in first-line and neoadjuvant settings [4].

The biology of TETs is still largely unknown and their etiology and pathogenesis still need to be clarified. Neither environmental nor genetic risk factors have been identified and few target-specific drugs have been developed [5].

A major challenge that has hampered preclinical studies is the paucity of preclinical models. The absence of relevant spontaneous and xenograft-based animal models has contributed to the lack of translational studies.

Patient-Derived Xenografts (PDXs) are state-of-the-art preclinical tools to investigate the efficacy of new treatments. PDX models reflect the heterogeneity of human tumors in terms of genomic alterations and gene expression profiles, and this increases their relevance for pre-clinical studies [6].

Here we report the establishment of two PDX models derived from two subsequent relapses of the same patient. The new PDXs were characterized for histology and mutation profile comparing the data with the patient’s samples.

## Main text

**Fig. 1:**
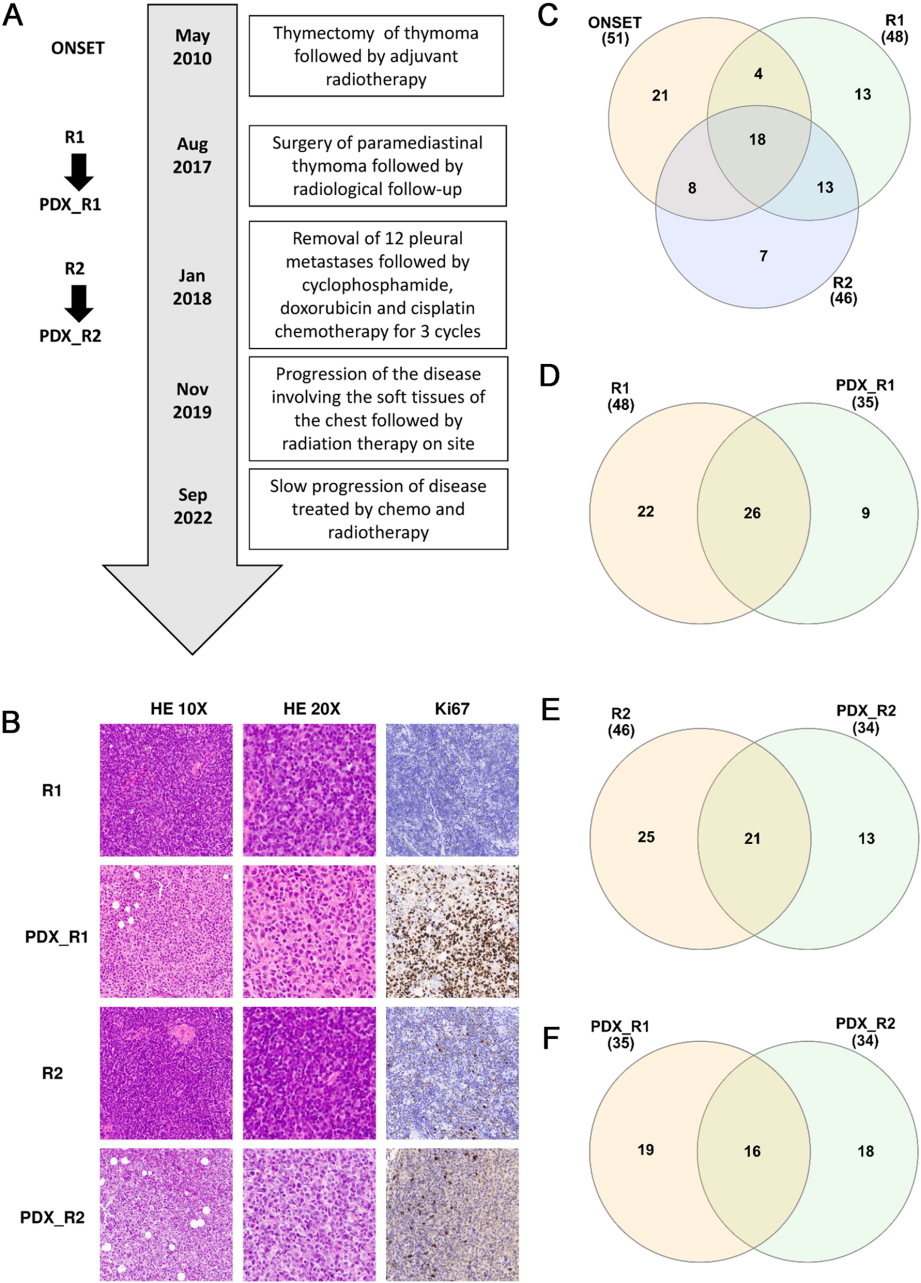
**A** Patient history showing major therapeutic interventions and time points of PDX establishment. **B** Histological analyses of R1, R2, PDX_R1 and PDX_R2 after hematoxylin/eosin and Ki67 staining. **C** Venn diagram showing the common and specific mutations between onset, R1 and R2. **D** Venn diagram showing the common and specific mutations between R1 and PDX_R1. **E** Venn diagram showing the common and specific mutations between R2 and PDX_R2. **F** Venn diagram showing the common and specific mutations between PDX_R1 and PDX_R2

The history of the patient from whom the xenografts were generated is depicted in Fig. 1A. A 31 year-old female with myasthenia gravis (with anti-AchR antibodies) on medical treatment was subjected in 2010 to *thymectomy en bloc* with a portion of pericardium. Histological examination revealed a B2 thymoma with infiltration into the thymic capsule and the mediastinal adipose tissue. The patient underwent adjuvant radiotherapy, with a total dose of 50.4 Gy in 25 fractions, on the thymic lodge. In 2017, the patient started complaining of cough and a sense of retrosternal weight. The CT scan revealed a paramediastinal mass, largely infiltrating the sternal body, the chondro-sternal joints, the xiphoid process and the pericardial fat. An 18-fluorodeoxyglucose PET scan identified hypermetabolic tissue only in the site of the known lesion. After multidisciplinary evaluation, in consideration of the potentially achievable radicality, surgery was proposed as the first approach. This consisted in removal of the sternal body, with the chondro-sternal joints from the third to the seventh bilaterally, the pericardial fat, and an atypical resection of the right upper pulmonary lobe, which was infiltrated. The histological examination confirmed a relapse of B2 thymoma. Radiation therapy was contraindicated because of the previous treatment, so the patient was kept on radiological follow-up. One year later a CT scan revealed multiple pleural and diaphragmatic localizations. The patient underwent a diagnostic and cytoreductive Video Assisted Thoracic Surgery procedure. The thoracoscopy confirmed the suspicion of relapse and 12 pleural metastases were removed. The surgery, however, was not radical because of the visceral pleura involvement. The patient underwent chemotherapy with cyclophosphamide, doxorubicin and cisplatin for 3 cycles. In November 2019, the patient presented with a progression of the disease involving the soft tissues of the chest wall and infiltrating the ninth right rib. She was referred for radiation therapy on that site. At last follow-up in September 2022 the patient was alive, with slow progression of disease treated by chemo- and radiotherapy.

We collected tumor samples from the patient after the surgery of the two relapses (R1 and R2), and transplanted them in Rag2-Il2rg double knockout immunodeficient mice to generate PDX. We were able to generate PDX from both samples, named PDX_R1 and PDX_R2. Both models were passed for seven passages with a take of 87% for PDX_R1 and 58% for PDX_R2. The PDXs were evaluated for the time needed to reach 500 mg, which was respectively 30 ± 10 and 36 ± 7 days for PDX_R1 and PDX_R2.

The PDX_R1 and PDX_R2 collected at passage IV were formalin-fixed and paraffin embedded for histological analysis. Consecutive 4-μm thick sections were cut from each tissue block and stained with hematoxylin and eosin or used for immunohistochemical analyses. The two PDXs showed preserved morphology when stained with hematoxylin and eosin. The tumors were composed of neoplastic epithelial cells with a scattered plump shape with vesicular nuclei and distinct nucleoli. Foci of necrosis were also detected which were not identified in the original tumors, and there was a higher mitotic count. Slides were processed with the automatic system BenchMark XT (Ventana Medical Systems) for immunohistochemical evaluation. Reactions, revealed using UltraViewTM Universal DAB, showed no staining for Cytokeratins AE1/AE3 in either PDX while patient tumors were positive (data not shown). Semiquantitative determination of the Ki67 labeling index was nearly 70%, indicating intense proliferating activity in the PDXs. Representative images of the different staining are reported in Fig. 1B.

Genomic DNA was extracted from FFPE tissue of the onset tumor (T), the two relapses (R1 and R2) and the two PDXs (PDX_R1 and PDX_R2) at passage IV for sequencing using a custom panel of genes (Table S1) selected for their frequency of mutations in most common tumor types. DNA were used for library preparation and sequenced using a Illumina-V2 cartridge for MiSeq (Illumina).

A first de-multiplexing step prepared data for quality control. We produced quality reports with FastQC (http://www.bioinformaticsbabrahamacuk/projects/fastqc/), which identified subthreshold sequence fragments. The following trimming procedure removed the marked areas: fastp FASTQ preprocessor filtered them out, while also providing a supplementary report confirming the FASTQ files’ quality. Read mapping was done using BWA-MEM [7]. Human and PDX samples were mapped against both UCSC Human HG38 and UCSC Mouse MM39 reference genomes (https://hgdownload.soe.ucsc.edu/downloads.html). The resulting SAM files, sorted and converted to BAM file format, underwent computational deconvolution with XenofilteR [8], which sorted out mouse reads from the input data. We adopted GATK best practices workflow (https://gatk.broadinstitute.org/hc/en-us/sections/360007226651-Best-Practices-Workflows) for the whole variant calling procedure. After cleaning BAM files and Base Quality Score Recalibration, we obtained the somatic variants VCF file using Mutect2 [9]. We then annotated the variants employing ANNOVAR [10]. Single-sample annotated files were merged into one table for variant analysis.

Considering the non-synonymous mutations and the base changes in the 5’UTR and 3’UTR with an allelic frequency higher than 0.2, we detected 51 mutations in the onset sample (Table S2), 48 in the first relapse (Table S3), 46 in the second relapse (Table S4), 35 in the PDX_R1 (Table S5) and 34 in PDX_R2 (Table S6). Eighteen mutations were common to all three of the patient’s samples (35.3, 37.5 and 39.1% respectively at onset, R1 and R2). In addition, four mutations were common at onset and R1 relapse, eight mutations were common at onset and R2 relapse and thirteen mutations were common at R1 and R2 relapses. Twenty-one (41.1%), 13 (27.1%) and 7 (15.2%) mutations were specific for the onset, first relapse and second relapse respectively. Figure 1C is a graphic representation of the common and specific mutations among the patient’s samples. The PDX_R1 shared 26 (74.3%) mutations with the tumor from which it derived while respectively 22 and 9 mutations were specific for the relapse and PDX_R1 (Fig. 1D). Of the 34 mutations of PDX_R2, 21 (61.8%) were shared with the relapse R2. The PDX_R2 and R2 harbored respectively 13 and 25 additional specific mutations (Fig. 1E). The two PDXs had 16 mutations in common (respectively 45 and 47% of PDX_R1 and PDX_R2) (Fig. 1F). The five samples analyzed shared the 11 mutations reported in Table 1.

**Table 1:** Mutations shared among all samples

GENE	CHROMOSOME	START POSITION	TYPE	BASE CHANGE	AA CHANGE
MAML2	chr11	96,092,130	exonic	c.G1901A	p.S634N
NF1	chr17	31,330,458	exonic	c.A5709C	p.L1903F
ERBB2	chr17	39,723,955	exonic	c.C2252G	p.A751G
TP53	chr17	7,676,483	UTR5		
TET2	chr4	105,235,030	exonic	c.C1088T	p.P363L
TET2	chr4	105,243,584	exonic	c.C3609G	p.S1203R
FBXW7	chr4	152,322,813	UTR3		
APC	chr5	112,707,571	UTR5		
APC	chr5	112,841,059	exonic	c.T5411A	p.V1804D
APC	chr5	112,843,804	exonic	c.A8156C	p.E2719A
AFDN	chr6	167,965,955	exonic	c.A5017C	p.K1673Q

## Conclusions

This study reports the isolation and characterization of two newly established PDX derived from two relapses of the same patient. This is a substantial achievement not only because it is the first PDX model ever derived from a thymoma, but also because it recapitulates faithfully both the molecular characteristics and evolution of the thymoma it derives from. With these characteristics, it will be possible to understand both the tumor evolution and changes in therapy response better.

From the histological point of view, both morphological and immunohistochemical results suggest a sort of “de-differentiation” of the lesions of the PDXs. In both PDXs, the epithelial elements showed increased pleomorphism and several atypical mitotic figures compared to the original tumors. In addition, foci of necrosis, not identifiable in the primary lesions, were evident in PDXs. The presence of necrosis only in PDXs might be explained by the higher proliferative rate of the models compared to the patient’s tumors.

Starting from analysis of the tumor at onset, the number of mutations is not particularly high, in agreement with the few data in literature. Looking at the evolution (i.e. mutations at R1 and R2) a significant proportion of the original mutations are present also at the relapses, indicating that these are likely to contain driver mutations necessary for tumor expansion. Conversely there are some mutations only at relapse that might be (although this at present is only a speculation) additional mutations favoring tumor growth. This hypothesis is in line with the short time between R1 and R2 (5 months) while the first relapse took years to occur.

Importantly, the two PDX, derived from R1 and R2 maintain some mutations present in the human tumor of origin thus enabling us to address the role of these mutations in experimental models. Since the onset of the thymoma was more than 10 years earlier, unfortunately we could not have PDX from the original tumor, and this could potentially hamper the discovery of genes likely to play a role in fast tumor growth.

Nonetheless, these models are unique for this rare tumor and present an exceptional opportunity for a deeper understanding of the biology of the tumor, its progression and its response to new therapies.

## Supplementary Information

**Additional file 1: Table S1**. Genes included in the panel. **Table S2**. Mutations in the ONSET. **Table S3**. Mutations in the R1. **Table S4**. Mutations in the R2. **Table S5**. Mutations in the PDX_R1. **Table S6**. Mutations in the PDX_R2.

## Data Availability

The datasets used and/or analysed during the current study are available from the corresponding author on reasonable request.

## Abbreviations

TET: Thymic epithelial tumor
WHO: World Health Organization
FFPE: Formalin-fixed and paraffin-embedded
PDX: Patient-derived xenografts
R1: Relapse 1
R2: Relapse 2
PDX_R1: Patient-derived xenografts of relapse 1
PDX_R2: Patient-derived xenografts of relapse 2
